# A highly predictive cardiac positron emission tomography (PET) risk score for 90-day and one-year major adverse cardiac events and revascularization

**DOI:** 10.1007/s12350-022-03028-y

**Published:** 2022-12-19

**Authors:** Raymond O. McCubrey, Steve M. Mason, Viet T. Le, Daniel L. Bride, Benjamin D. Horne, Kent G. Meredith, Nishant K. Sekaran, Jeffrey L. Anderson, Kirk U. Knowlton, David B. Min, Stacey Knight

**Affiliations:** 1 Intermountain Medical Center Heart Institute, Intermountain Healthcare, 5121 Cottonwood St Bldg. 1 Floor 4, Murray, UT 84107 USA; 2 Division of Cardiovascular Medicine, Department of Medicine, Stanford University, Stanford, CA USA; 3 Department of Internal Medicine, School of Medicine, University of Utah, Salt Lake City, UT USA

**Keywords:** PET/CT, revascularization, risk score, major adverse cardiovascular events

## Abstract

**Background:**

With the increase in cardiac PET/CT availability and utilization, the development of a PET/CT-based major adverse cardiovascular events, including death, myocardial infarction (MI), and revascularization (MACE-Revasc) risk assessment score is needed. Here we develop a highly predictive PET/CT-based risk score for 90-day and one-year MACE-Revasc.

**Methods and results:**

11,552 patients had a PET/CT from 2015 to 2017 and were studied for the training and development set. PET/CT from 2018 was used to validate the derived scores (n = 5049). Patients were on average 65 years old, half were male, and a quarter had a prior MI or revascularization. Baseline characteristics and PET/CT results were used to derive the MACE-Revasc risk models, resulting in models with 5 and 8 weighted factors. The PET/CT 90-day MACE-Revasc risk score trended toward outperforming ischemic burden alone [*P* = .07 with an area under the curve (AUC) 0.85 vs 0.83]. The PET/CT one-year MACE-Revasc score was better than the use of ischemic burden alone (*P* < .0001, AUC 0.80 vs 0.76). Both PET/CT MACE-Revasc risk scores outperformed risk prediction by cardiologists.

**Conclusion:**

The derived PET/CT 90-day and one-year MACE-Revasc risk scores were highly predictive and outperformed ischemic burden and cardiologist assessment. These scores are easy to calculate, lending to straightforward clinical implementation and should be further tested for clinical usefulness.

**Supplementary Information:**

The online version contains supplementary material available at 10.1007/s12350-022-03028-y.

## 摘要

在40-70%有缺血症状的患者中, 有创冠状动脉造影检查未发现梗阻性冠状动脉疾病 (INOCA) 。当这一病变组接受冠状动脉功能学检查, 大约三分之二的人有明显的冠状动脉微血管功能障碍 (CMD), 这与不良预后独立相关。CMD有四种不同的表型或亚组, 每种表型都有独特的病理生理机制和对应的不同治疗策略。CMD临床表型包括微血管性心绞痛、血管痉挛性心绞痛、混合型 (微血管和血管痉挛) 和非心脏症状 (重新分类为非INOCA) 。冠状动脉血管运动障碍国际研究组 (COVADIS) 提出了标准化的诊断标准。临床医生和指南对这些疾病的认识也在不断提高。

CorMicA研究提倡 "功能性血管造影 "的概念以指导分层医学治疗。对CMD的检测可以采用侵入性或非侵入性的方式进行。治疗方法大致分为两类: 控制心血管风险和缓解心绞痛症状。患者治疗管理应个体化, 并定期重新评估疗效。基于表型的管理对患者和临床医生来说都是值得努力的, 这与 "精准医学 "以改善预后、症状改善和提高生活质量的概念相一致。在此, 我们对于INOCA患者提出了一种基于表型管理的现代方法。

## Introduction

Annually approximately 3.8 million patients undergo cardiac stress testing in the USA.^1^ As high as 15% of these patients will have a false-negative result and 2.4% of these misdiagnosed patients have a subsequent major adverse cardiac event (MACE).^2^ Cardiac PET/CT (positron emission tomography/computed tomography) may reduce these misdiagnoses by providing higher image quality.^3–10^ While PET has been around for decades, clinical use of cardiac PET/CT has been relatively limited. However, new radiopharmaceuticals and changes in reimbursement have contributed to the recent rapid growth of PET/CT.^11,12^ Like other imaging modalities, the comprehensive interpretation of PET/CT scans has a steep learning curve, especially in more complex or ambiguous cases.^13–16^ There is also inherent variability in the interpretation of cardiac images and cardiac risk assessment.^17–21^ However, developing a risk assessment score for MACE and revascularization (MACE-Revasc) using the large amount of data gathered (e.g., ischemic burden and myocardial blood flow) by PET/CT could help minimize the learning curve and reduce inter-operator variability in risk assessment.

The purpose of this study was to develop a PET/CT-based risk assessment score for 90-day and one-year MACE-Revasc outcomes. This was done using data from Intermountain Medical Center, which switched to a PET/CT-centric myocardial perfusion imaging center in 2013 and conducts about 4000-5000 cardiac PET/CT scans annually.^3,22^ Therefore, for the development of a PET-based risk assessment, we were able to have large training, development, and test data sets containing common, standard clinical PET/CT elements. A key focus in the development of the risk assessment score was to ensure that it was useful clinically. Consequently, the developed numeric score was based on assigned weights for categorical values of common clinical and PET/CT results. We compared the developed risk score to ischemic burden and the interpreting cardiologist assessment of risk as documented in the official clinical interpretation of the studies.

## Methods

This study was approved by the Intermountain Healthcare Institutional Review Board with a waiver of consent. Investigations were performed in accordance with the Declaration of Helsinki.

### Study population

All unique patients that completed a PET/CT study from January 1, 2015 to December 31, 2017 at Intermountain Medical Center were used for developing the risk score (n = 11,552). Intermountain Medical Center is the major referral hospital for Intermountain Healthcare, an integrated healthcare system of 24 hospitals and over 215 clinics. This study was restricted to 2015 and after due to the lack of electronic capture and coding of transient ischemic dilation (TID) before this period. This study population was split 70:30 into training (n = 7996) and development (n = 3526) sets.

A separate test set was used to assess the accuracy of the generated PET-based risk score algorithm. This test set contained all patients that had a clinically indicated PET/CT study from January 1, 2018 to December 31, 2018 at Intermountain Medical Center (n = 5049).

### PET/ CT myocardial perfusion imaging

PET/CT imaging was performed on a Siemens Biograph (LSO crystal, 3-dimensional list modes, with 16 slice CT) camera with rubidium-82 chloride (Rb-82). Weight-based Rb-82 dosage varied from 20 to 40 mCi for both rest and stress images. Rest and stress low-dose CT topograms and attenuation correction images are obtained with all PET scans for anatomic alignment of the PET images and for calibration of PET/CT data, respectively. Pharmacologic stress was achieved in all patients with regadenoson. Both gated rest and stress images were acquired and iteratively reconstructed using the manufacturer-recommended protocol. PET/CT images were analyzed using commercially available software packages (syngo.VIA, Siemens Healthineers, Malvern PA, and Corridor4DM, Invia Medical Imaging Solutions, Ann Arbor MI). A complete description of the PET/CT acquisition parameters is provided in the Supplementary File. The reading and interpretation of the PET/CT studies during the study timeframe were performed by cardiologists who were board certified in nuclear cardiology.

### Clinical and PET imaging data

The prior clinical diagnoses were based on a combination of patient self-report at the time of PET/CT and diagnosis coding in Intermountain Healthcare's electronic medical record. Smoking status was based on patient self-report at the time of PET/CT. As formal coronary artery calcification (CAC) quantified scoring was not done routinely on these patients, the presence or absence of CAC was determined using the cardiologist’s report of CAC present based on the low-dose attenuation correction CT images. The estimation of CAC using low-dose attenuation correction CT has been shown to correlate well with the estimation of CAC scores obtained as part of the standard Agatston score calculation.^23^ The mean global myocardial coronary flow reserve was used as the overall assessment of myocardial blood flow. TID was determined using syngo.via. Change in ejection fraction (EF) from rest to stress periods was calculated. The ischemic burden was determined using the difference between the summed rest and summed stress scores from 17 segments and then dividing this by 68.^24^ As part of the electronic generated PET/CT report, the risk of a short-term ischemic event (low, moderate, or high) was recorded by the reading cardiologist. While the cardiologist would make this assessment using the measured PET/CT parameters and clinical characteristics, there was no set algorithm or protocol for determining these risk levels. Therefore, the cardiologist’s risk assessment was subjective.

### Endpoints

Major cardiovascular events of all-cause death, myocardial infarction (MI), and revascularization were studied for both 90-day and one-year periods (MACE-Revasc). All-cause death was determined using hospital discharge status and death certificate records from the state of Utah. The cause of death was not available as part of these reports. Intermountain Healthcare’s hospital diagnoses and elevation in troponin were used to determine subsequent MI. Revascularization (percutaneous coronary intervention and/or coronary artery bypass grafting) was based on hospital procedure billings and coronary catheterization reports. We choose to include revascularization in our MACE-Revasc model despite this outcome being driven by the physician’s interpretation and is not a hard event. We realize the outcome could be biased because of this. However, this bias will be present for the other two risk assessments used for comparisons (i.e., ischemic burden and physician risk prediction). Furthermore, a major purpose of a stress test is to determine those individuals with ischemia and for whom revascularization is needed. Therefore, the inclusion of revascularization in the outcome was deemed to be important. We did, however, conduct a sensitivity analysis in which we examined our derived risk scores prediction power for MACE without revascularization (i.e., death and non-fatal MI).

### Development of risk score

**Figure 1:**
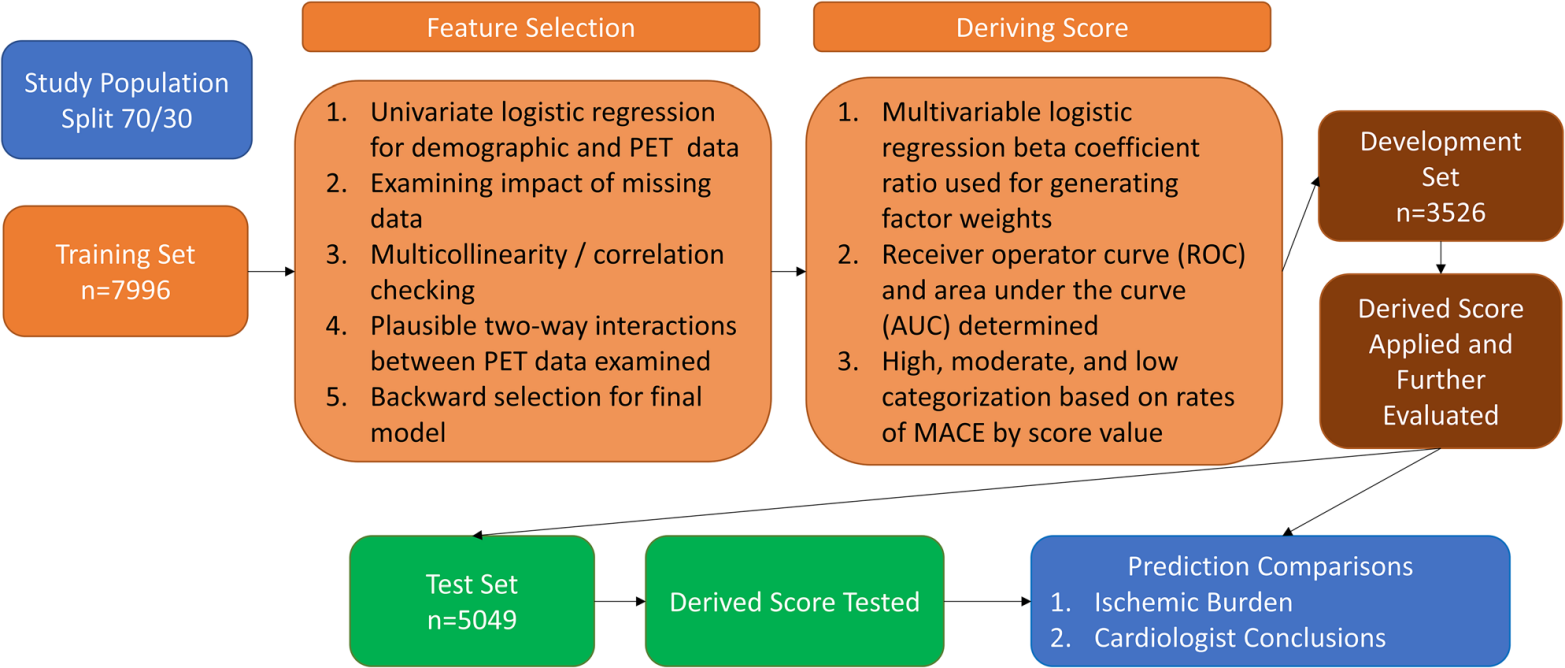
PET/CT Risk Score Development Diagram

The basic flow of the score development is outlined in Fig. 1. The study population was split 70:30 into a training and development set (dev. set). Continuous factors were categorized using existing and commonly used thresholds for change in EF (< 3%, 3%-4%, ≥ 5%) and ischemic burden (< 5%, 5%-10%, > 10%). The cut-offs for coronary flow reserve (CFR) are not established and have been suggested to be “generally arbitrary and may vary slightly between labs, software used, stressors used, and published studies.”^25^ Based on our experience with the use of regadenoson, we chose a cut-off of > 2.3 for normal, 1.5-2.3 as abnormal, and < 1.5 as highly abnormal. Given that regadenoson has been shown to achieve only 80% of dipyridamole stress perfusion,^26^ these values would correspond to CFR of about > 1.8, 1.8-1.2, and < 1.2 when using dipyridamole. These cut-offs would be very similar to the cut-offs for normal (or mildly abnormal), abnormal, and highly abnormal as suggested by others.^25^ Based on our prior research experience with TID for regadenoson, the thresholds used for TID were ≤ 1.0 for normal, 1.01-1.10 for abnormal, and ≥ 1.10 for highly abnormal.^27^

While less than 8% of the data were missing for any given factor (see supplementary table 1), we examined the impact of missing values by adding a category of “missing” to the factor. The prediction for the missing values was similar to the reference category for all the factors. This indicated that “missing” would not impact the final scoring system and thus, no imputation of the missing values was done.

Using the training set, we determined the demographic, clinical, and PET result factors that were univariately significantly (*P* < .05) associated with the outcomes. For those with a significant association, we checked for multicollinearity and pairwise correlation. A meaningful pairwise correlation was defined as a statistically significant correlation above ≥ 0.30. When detected, the factor with the largest AUC value for the outcome was kept. In the case that a factor was correlated with multiple other factors and did not have the largest AUC for all comparisons, it was eliminated in favor of keeping the model parsimonious (see Supplementary Table S2 for factor selection details). Clinically plausible two-way interactions from the PET/CT results, including ischemic burden by transient ischemic dilation and ischemic burden by ejection fraction, were also considered in the model building process. These uncorrelated factors and interactions were included in a backward selection logistic regression model with selection based on AIC (Akaike Information Criterion). From this analysis, several factors including the interactions were eliminated. Once the final model was determined, we used the beta coefficients for significant factors and the ratios of these to generate the weighted scores for each factor in the final models (see supplementary tables S3a and S3b for the final logistic models and beta coefficients).

To qualify the risk associated with a score, we have determined thresholds for low, moderate, and high risk for the 90-day and one-year MACE-Revasc risk. The threshold between high and moderate risk was set where the specificity of the score is ≥ 90%, and the threshold between moderate and low risk was set where the sensitivity is ≥ 90%. This approach tends to provide an appropriate distribution in all three categories and has been used previously in setting thresholds for similarly derived risk scores.^28^ Finally, we determined the Brier score^29^ for our derived risk scores using the training set and the predictive probability for our derived score values (based on logistic regression). The Brier score ranges from 0 to 1, with 0 indicating a perfect model prediction and 1 indicating no predictive value of the model. Thus, lower Brier scores indicate more accurate predictions.

### Statistical comparisons

Our derived PET/CT risk scores were compared to both the ischemic burden and the cardiologist’s reported risk for the prediction of 90-day MACE-Revasc and one-year MACE-Revasc. Receiver operating characteristic (ROC) curves were generated for the training, development, and test sets, and the areas under the curve (AUC) for the continuous score values compared to the continuous ischemic burden values. The significance of this comparison was done using the DeLong test. Additional continuous comparisons with CRF and summed stress scores were done. Finally, the cardiologist reading the PET/CT-reported risk (low, moderate, and high) at the time of the scan was compared to the derived scores categorized as low, moderate, and high, using the method described above. Net classifications for events and non-events were calculated for this comparison using low/moderate risk compared to high risk. We used bootstrapping to determine the 95% confidence interval for these reclassifications. All models and testing were done using R (version 4.0.3) and the non-standard packages used included pROC (for AUC calculations) and rap (for NRI confidence intervals).

## Results

### Study population characteristics and MACE-Revasc outcomes

The patient and clinical characteristics for the three study populations are shown in Table 1. In general, the PET/CT patients were on average 65 years old, just over half were male, many had risk factors for coronary artery disease, and over a quarter had a prior history of MI or revascularization. While there existed statistically significant differences between the sets, the largest and clinically significant difference was in the decrease in a history of coronary artery disease for the test set compared to the other two sets (57% vs 77%). The PET/CT results for the three groups are shown in Table 2. For all three sets, about 10% of the patients had an ischemic burden > 10% and about 5% were at high risk based on the cardiologist’s assessment. There was a slight decrease in the percentage of patients without CAC in the test set compared to the other two sets (25% vs 31%).

**Table 1:** Patient and clinical characteristics for study population sets

	Training	Development	Test	*P*-value^a^
	7996	3526	5049	
Age categories				.12
< 50	931 (11.6%)	403 (11.4%)	535 (10.6%)	
50–59	1592 (19.9%)	722 (20.5%)	955 (18.9%)	
60–69	2431 (30.4%)	1013 (28.7%)	1593 (31.6%)	
70–79	2036 (25.5%)	932 (26.4%)	1310 (25.9%)	
80+	1006 (12.6%)	456 (12.9%)	656 (13%)	
Gender				.02
Male	4452 (55.7%)	1906 (54.1%)	2884 (57.1%)	
Female	3544 (44.3%)	1620 (45.9%)	2165 (42.9%)	
Race				.008
White	7434 (93.0%)	3274 (92.9%)	4607 (91.2%)	
Non-White	562 (7.0%)	252 (7.1%)	442 (8.8%)	
BMI categories				.33
Underweight (< 18.5)	86 (1.1%)	45 (1.3%)	54 (1.1%)	
Normal (18.5–25)	1516 (19%)	605 (17.2%)	932 (18.5%)	
Overweight (25–30)	2474 (30.9%)	1107 (31.4%)	1601 (31.7%)	
Obese (30+)	3920 (49%)	1769 (50.2%)	2462 (48.8%)	
Patient type				< .0001
Emergency or outpatient	6279 (78.5%)	2795 (79.3%)	4265 (84.5%)	
Inpatient	1717 (21.5%)	731 (20.7%)	784 (15.5%)	
Smoking history				.0001
Never	4905 (61.3%)	2220 (63.0%)	3094 (61.3%)	
Former	2096 (26.2%)	907 (25.7%)	1304 (25.8%)	
Current	659 (8.2%)	230 (6.5%)	363 (7.2%)	
Unknown	336 (4.2%)	169 (4.8%)	288 (5.7%)	
Diabetes	3342 (41.8%)	1544 (43.8%)	2087 (41.3%)	.06
Hypertension	6710 (83.9%)	2996 (85%)	4145 (82.1%)	.0010
Hyperlipidemia	6269 (78.4%)	2801 (79.4%)	3841 (76.1%)	.0004
Prior MI	1390 (17.4%)	674 (19.1%)	830 (16.4%)	.01
History of CAD	6117 (76.5%)	2730 (77.4%)	2852 (56.5%)	< .0001
Prior Revascularization	2299 (28.8%)	1108 (31.4%)	1387 (27.5%)	.0003

*BMI*, body mass index; *MI*, myocardial infarction; *CAD*, coronary artery disease

^a^*P* values are based on chi-square (categorical variables) and ANOVA (continuous variables) tests

**Table 2:** PET/CT results for the study populations

	Training	Development	Test	*P*-value
	7996	3526	5049	
CAC				< .0001
Absent	2535 (31.7%)	1080 (30.6%)	1279 (25.3%)	
Present	5461 (68.3%)	2446 (69.4%)	3770 (74.7%)	
Change in EF				< .0001
≥ 5%	4699 (58.8%)	2114 (60.0%)	3290 (65.2%)	
3–4%	1321 (16.5%)	515 (14.6%)	752 (14.9%)	
< 3%	1976 (24.7%)	897 (25.4%)	1007 (19.9%)	
CFR				< .0001
> 2.3	3231 (40.4%)	1389 (39.4%)	2179 (43.2%)	
1.5–2.3	3338 (41.7%)	1515 (43.0%)	2120 (42.0%)	
< 1.5	1427 (17.8%)	622 (17.6%)	750 (14.9%)	
TID				< .0001
≤ 1.0	4084 (51.1%)	1772 (50.3%)	2769 (54.8%)	
1.0–1.10	2212 (27.7%)	1017 (28.8%)	1564 (31.0%)	
> 1.10	1700 (21.3%)	737 (20.9%)	716 (14.2%)	
Ischemic burden				.85
< 5	6586 (82.4%)	2894 (82.1%)	4178 (82.7%)	
5–10	621 (7.8%)	287 (8.1%)	398 (7.9%)	
> 10	789 (9.9%)	345 (9.8%)	473 (9.4%)	
Cardiologist conclusions				.39
Low risk	6567 (82.1%)	2887 (81.9%)	4198 (83.1%)	
Moderate risk	982 (12.3%)	431 (12.2%)	596 (11.8%)	
High risk	447 (5.6%)	208 (5.9%)	255 (5.1%)	

*CAC*, coronary artery calcium; *EF*, ejection fraction; *CFR*, coronary flow reserve; *TID*, transient ischemic dilation

The 90-day and one-year MACE-Revasc outcomes for the study populations are shown in Table 3. The rate of 90-day MACE-Revasc was about 6% for the sets, most of this driven by revascularization within 90 days. The one-year MACE-Revasc ranged from 13% to 16%, and most of these were revascularizations, followed by myocardial infarctions and deaths.

**Table 3:** MACE-Revasc outcomes for the study population sets

	Training	Development	Test	*P*-value
	7996	3526	5049	
90-day outcomes				
MACE-Revasc	510 (6.4%)	206 (5.8%)	367 (7.3%)	.02
Death	105 (1.3%)	45 (1.3%)	53 (1%)	.39
MI	154 (1.9%)	52 (1.5%)	76 (1.5%)	.10
Revascularization	292 (3.7%)	127 (3.6%)	267 (5.3%)	< .0001
One-year outcomes				
MACE-Revasc	1262 (15.8%)	532 (15.1%)	640 (12.7%)	< .0001
Death	217 (2.7%)	77 (2.2%)	108 (2.1%)	.07
MI	604 (7.6%)	247 (7%)	205 (4.1%)	< .0001
Revascularization	594 (7.4%)	278 (7.9%)	378 (7.5%)	.68

*MI*, myocardial infarction; MACE-Revasc is a composite of death, MI, and revascularization

### PET/CT 90-day MACE-Revasc risk score

**Figure 2:**
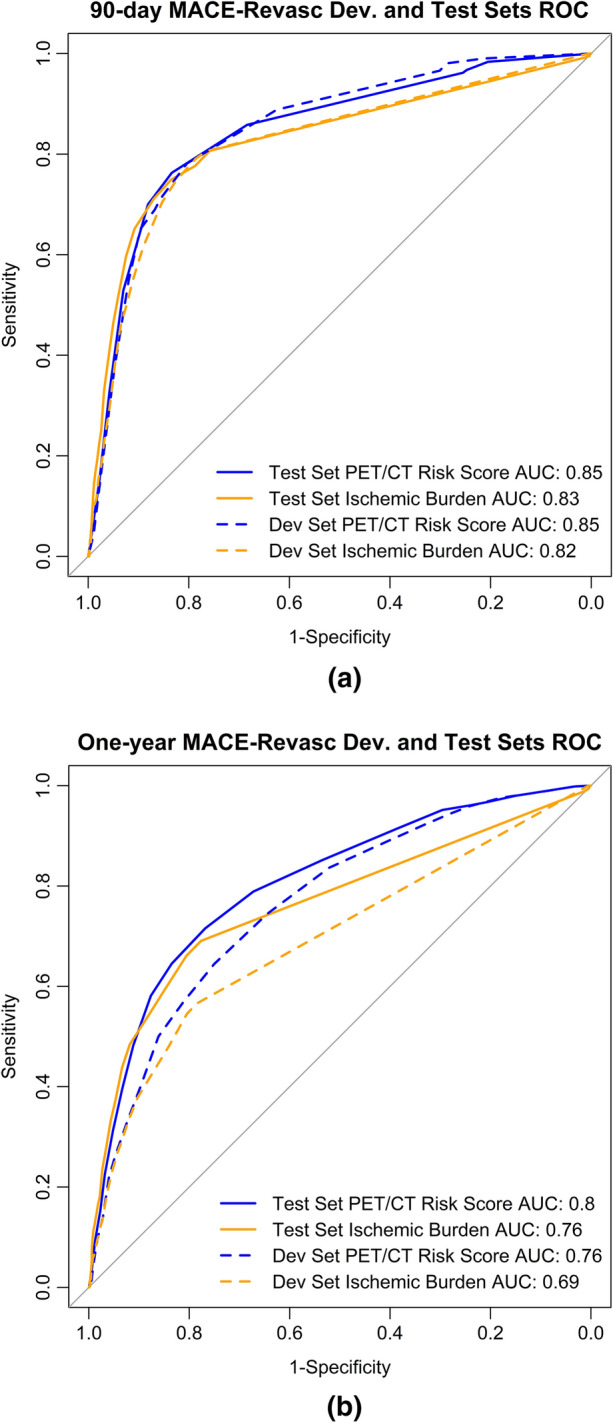
90-day (**a**) and One-Year (**b**) MACE-Revasc PET/CT Risk Score vs Ischemic Burden Receiver Operating Characteristic Curve (ROC) for the development (dev) and test sets. *AUC*, area under the curve; *ROC*, receiver operating characteristic curve; *Dev*, development

Based on the logistic regression factor selection and modeling, the PET/CT 90-day MACE-Revasc risk score and factor weights are shown in Table 4. No interactions made it into the final score due to simpler models having better AIC during the model selection process. Ischemic burden was the major contributor to the developed PET/CT 90-day MACE-Revasc risk score. Therefore, the overall prediction for the PET/CT 90-day MACE-Revasc risk score was only slightly better than the use of ischemic burden (*P* = .01 development set/*P* = .07 test set); the area under the curve for the PET/CT 90-day MACE-Revasc risk score was 0.85 for both the development and test sets compared to 0.82 and 0.83, respectively, for ischemic burden alone (Fig. 2a). The summed stress scores had a similar AUC (test set AUC 0.83) and the CFR had a significantly lower AUC (test set AUC 0.83) compared to the PET/CT 90-day MACE-Revasc risk score (Supplementary Table S4). The Brier score for the PET/CT 90-day MACE-Revasc risk score was 0.14 and 0.15 for the development and the test sets, respectively.

**Table 4:** PET/CT Risk Score for 90-Day MACE-Revasc

Factor	Score
Ischemic burden > 10	6
Ischemic burden 5–10	4
CAC present	3
CFR < 1.5	1
TID > 1.1	1
Inpatient	1

Summed values range from 0 to 12, with values 0-3 = low risk, 4-7 = moderate, 8-12 = high risk

*CAC*, coronary artery calcium; *CFR*, coronary flow reserve; *TID*, transient ischemic dilation

**Figure 3:**
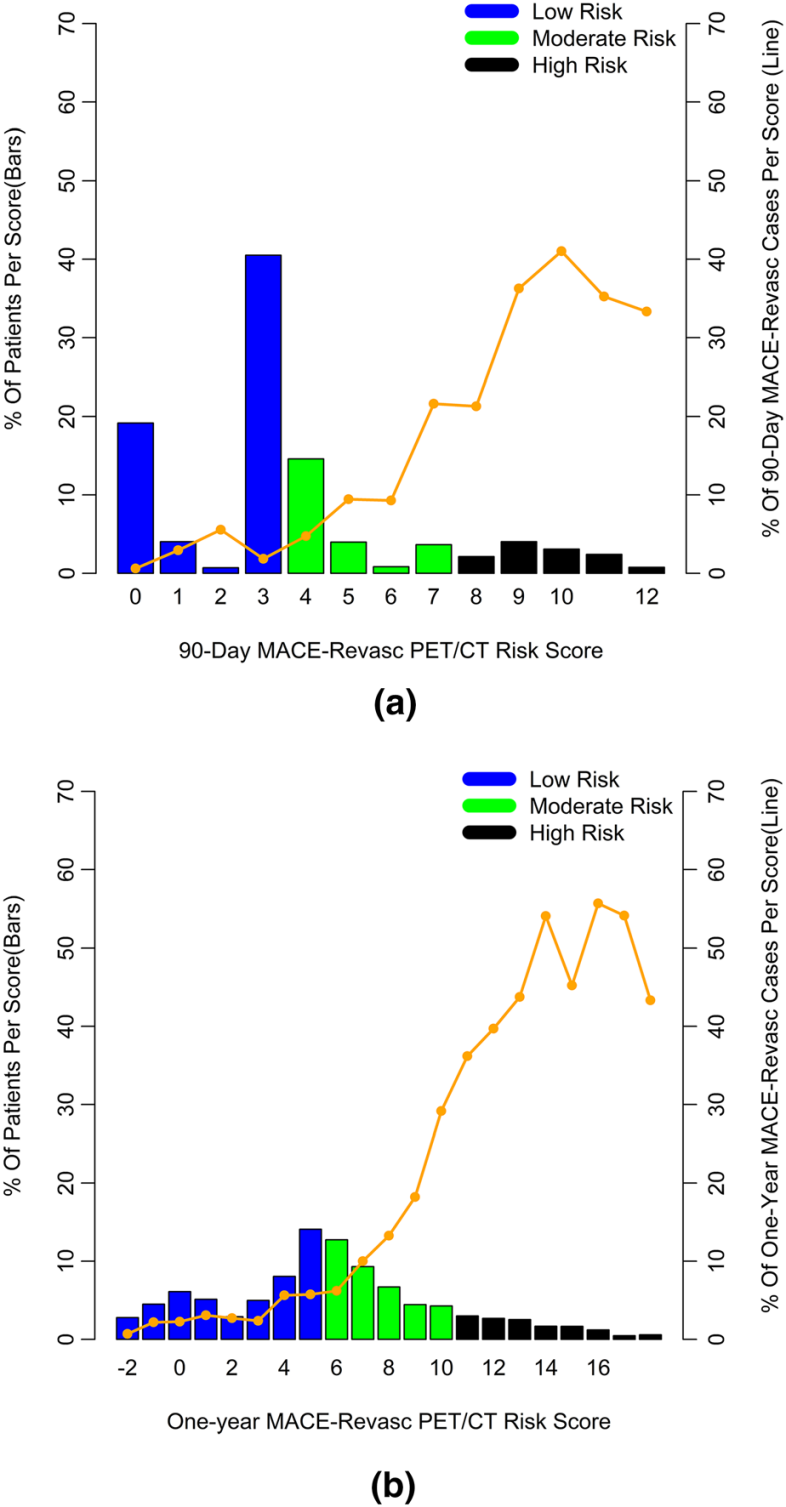
PET/CT 90-day MACE-Revasc Risk Score (bars) and Percent of 90-day MACE-Revasc Outcomes (line) in the Test set (**a**). Also, PET/CT One-Year MACE-Revasc Risk Score (bars) and Percent of One-Year MACE-Revasc Outcomes (line) in the Test set (**b**)

**Figure 4:**
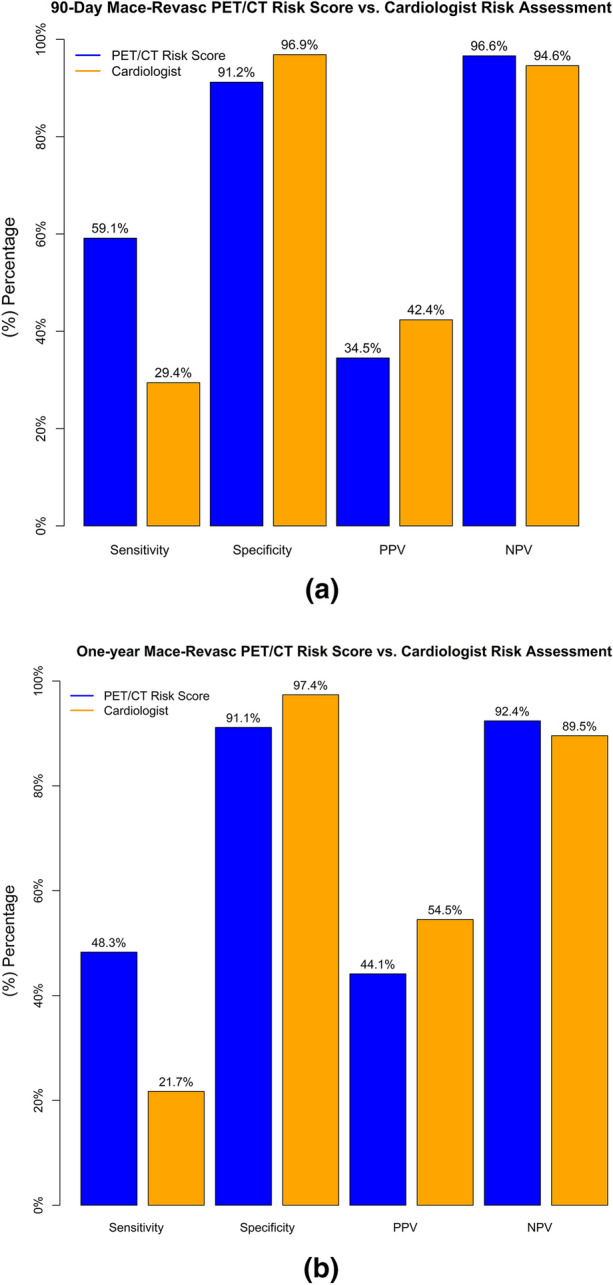
Prediction for the PET/CT 90-day MACE-Revasc Risk Score (**a**) & PET/CT One-Year MACE-Revasc Risk Score (**b**) to the cardiologist’s assessment of risk

The PET/CT 90-day MACE-Revasc risk score values and the percentage of MACE-Revasc events in the test set are shown in Fig. 3. Low risk was classified as a PET/CT 90-day MACE-Revasc risk score < 4, 4-7 as moderate risk, and > 7 as high risk. Using the test data, the rates of MACE-Revasc for these three groups were 1.0%, 1.9%, and 4.3%, respectively. The comparison in prediction for the PET/CT 90-day MACE-Revasc risk score to the cardiologist’s assessment of risk is shown in Fig. 4a. The net-reclassification index, using low/moderate risk versus high risk, was 24% (95% CI 19%, 30%) and indicated a significant improvement provided using the derived PET/CT 90-Day MACE-Revasc risk score prediction (Supplementary Table S5). In patients with 90-day events (n = 367), this reclassification would result in 120 (33%) having a higher PET/CT risk score compared to the cardiologist. However, in patients without events (n = 4682), the reclassification resulted in the incorrect increase in risk for 278 (6%) compared to the cardiologist.

### PET/CT one-year MACE-Revasc risk score

Based on the logistic regression factor selection and modeling, Table 5 shows the PET/CT one-year MACE-Revasc risk score. No interactions made it into the final score due to simpler models having better AIC during the model selection process. Ischemic burden and CAC presence were major contributors to the developed PET/CT one-year MACE-Revasc risk score. Other factors such as coronary flow reserve, smoking, and inpatient status were significant contributors to increased risk and obesity had a protective effect. The overall prediction for the PET/CT one-year MACE-Revasc risk score was better than the use of ischemic burden alone (*P* < .0001 dev and *P* < .0001 test); the area under the curve for the one-year PET/CT risk score was 0.76 for the development set and 0.80 for the test set, compared to 0.69 and 0.76 for ischemic burden alone, respectively (Fig. 2b). The summed stress scores (test set AUC 0.78) and the CFR (test set AUC 0.69) had significantly lower AUCs compared to the PET/CT one-year MACE-Revasc risk score (Supplementary Table S4). The Brier score for the PET/CT one-year MACE-Revasc score was 0.21 and 0.20 for the development set and the test set, respectively.

**Table 5:** PET/CT risk score for one-year MACE-Revasc

Factor	Score
Ischemic burden > 10	6
Ischemic burden 5–10	4
CAC present	5
CFR < 1.5	3
CFR 1.5–2.3	1
TID > 1	1
Current smoker	2
Inpatient	2
Diabetic	1
Obese (30 + BMI)	− 2

Summed value range from − 2 to 20, with values – 2 to 5 = low risk, 6-10 = moderate, 11-20 = high risk

*BMI*, body mass index; *CAC*, coronary artery calcium; *CFR*, coronary flow reserve; *TID*, transient ischemic dilation

The PET/CT one-year MACE-Revasc risk score values and the percentage of MACE-Revasc events in the test set are shown in Fig. 3b. Low risk was classified as a one-year MACE-Revasc score < 6, 6-11 as moderate risk, and ≥ 11 as high risk. Using the test data, the rates of MACE-Revasc for these three groups were 1.9%, 4.7%, and 6.1%, respectively. The comparison in prediction for the PET/CT one-year MACE-Revasc risk score to the cardiologist assessment of risk is shown in Fig. 4b. The net-reclassification index was 20% (95% CI 16%, 25%) and indicated a significant improvement provided using the score risk prediction (Supplementary Table S5). In patients with one-year events (n = 640), this reclassification would result in 181 (28%) having a higher risk score compared to the cardiologist. However, in patients without events (n = 4409), the reclassification resulted in the incorrect increase in risk for 291 (7%) compared to the cardiologist.

### PET/CT risk scores for prediction of MACE without revascularization

Using the test set, we applied the PET/CT 90-day and one-year risk scores to predict MACE (death and MI) without revascularization. Compared to the MACE-Revasc AUC, the MACE without revascularization included had decreased AUC values for 90-day and one-year 0.72 and 0.74, respectively (Supplementary Table S6). These AUC values were still larger than those associated with the ischemic burden (AUC 0.67) and CFR (AUC 0.67) for the 90-day MACE and the ischemic burden (AUC 0.65), CFR (AUC 0.69), and summed stress (AUC 0.69) for the one-year MACE (Supplementary Table S6). The PET/CT risk score had a significantly larger AUC (*P* < .05) for the 90-day MACE compared to ischemic burden and CFR and for the one-year MACE compared to ischemic burden, summed stress, and CFR.

## Discussion

We have derived a PET/CT 90-day MACE-Revasc risk score and a PET/CT one-year MACE-Revasc risk score that are highly predictive of events. These scores had statistically significant, although moderate, improvement over ischemic burden alone and the cardiologist’s assessment of risk. Both contained less than 10 factors and were based on summing integer values. Thus, these risk scores allow for easy implementation into practice.

Both the PET/CT 90-day and one-year MACE-Revasc risk scores were highly predictive of events with an accuracy measured by the area under the ROC curve of 0.85 and 0.80, respectively. While there have been limited numbers of risk scores built for populations undergoing PET/CT evaluation, our risk scores do perform better or similar than risk scores developed for stress testing patients and patients undergoing evaluation for chest pain. The Duke Treadmill Score has a similar accuracy (AUC 0.85 for 4-year death) to our PET/CT MACE-Revasc risk scores.^30^ However, the Duke Treadmill Score was created for patients without known coronary artery disease and our score comprises all patients being evaluated for coronary artery disease by PET/CT. In a recent study of risk scores in Emergency Department patients with chest pain, the HEART Score had the highest accuracy (AUC 0.77), followed by the TIMI risk score (0.73), GRACE (0.61), and EDACS (0.63).^31^ Our PET/CT risk scores appear to have greater predictive ability than these, but further evaluation of our score in different populations is needed.

The assessment of MACE risk from a PET/CT scan has routinely been based on ischemic burden.^32^ In clinical guidelines, it has been suggested that increases in ischemic burden have led to the re-evaluation of the medical regimen or the interventional plan.^33^ We did compare our risk score to ischemic burden and found a marginal, statistically significant difference for 90-day and a greater difference for our one-year MACE-Revasc risk assessment. As ischemic burden is the highest weighted factor in our risk scores, a marginal increase in 90-day risk is expected. However, the addition of other factors into our risk scores does improve the prediction, particularly for one-year outcomes. This is particularly evident in in the test set. This set had different clinical characteristics than the development set and while the ischemic burden distribution remained similar, the distributions of other PET results were different. Thus, because our risk score incorporates other factors it outperformed the ischemic burden in the test set.

The second most important factor in our risk scores was CAC, particularly in the one-year risk score, where CAC was almost as predictive as an ischemic burden. CAC has been shown to associate with MACE events.^34–36^ When added to existing risk scores, including ASCVD, CACS, and MESA, it has also been shown to improve risk prediction for MACE and revascularization.^37^ Therefore, CAC being the second most important factor in the risk scores is not surprising and does not require additional clinical testing as it is already incorporated into a cardiac PET/CT.

Prior machine learning studies have found that the use of other factors related to functional and perfusion data, besides ischemic burden, from cardiac myocardial perfusion imaging scans, increased predictive accuracy for MACE.^38,39^ We also found that coronary flow reserve and TID played moderate roles in our risk scores. Coronary flow reserve is a surrogate of fractional flow reserve, which has been found to be helpful for driving intervention decision-making regarding the revascularization of stenotic lesions.^40^ Thus, adding coronary flow reserve in the model, we believe, increases the effectiveness of predicting the revascularization component of our outcome. In addition, increases in TID ratios have been shown to be associated with increased risk of death^41^ and revascularization.^42^

Finally, as we developed these risk scores for all PET patients, the use of a factor to indicate whether the patient was currently an inpatient, improved discrimination for both 90-day and one-year MACE-Revasc outcomes. This is most likely a good factor to indicate overall health status. Similarly, smoking and diabetes were good predictors for poor one-year outcomes. Perhaps counterintuitively, obesity provided some protection for one-year MACE, but this is most likely due to the well-known obesity paradox for cardiovascular diseases. Many studies have shown that increased body mass index puts one at risk for cardiovascular disease, but provides a better prognosis for disease.^43^

Our risk scores, which combine and weight all these factors, could help cardiologists when assessing risks. We have shown that when compared to a cardiologist’s, our risk scores lead to a net reclassification of nearly a quarter for 90-day and 20% for one-year MACE-Revasc. This was mostly due to upward shifts in risks, which resulted in the risk score predicting more of the events. This is also reflected in the sensitivity of 59% for our 90-day and 48% for one-year events compared to 29% and 21%, respectively, for the cardiologist assessment. This increase occurred with little impact on specificity. A major reason for these differences is that the cardiologist assessment is focused more on the risk of an ischemic event and less on all-cause mortality risk, which was included in our outcome. However, as the overall health risk for a patient is important, using these risk scores might flag additional concerns for the cardiologist and perhaps drive additional assessment, treatment, and care.

Risk scores have been used in cardiovascular patient settings to drive better patient outcomes. In a small pilot study of heart failure patients where patients were randomized to have daily prediction scores versus a group given standard treatment, the group with the daily risk scores had a significant decrease in 30-day mortality and an increase in home discharges.^44^ Similarly, a larger study of heart failure inpatients found that a risk score-guided multidisciplinary team-based care process decreased 30-day readmission and mortality.^45^ An advantage to the implementation of our risk scores into clinical practices is the simplicity of collection and calculation of the scores. Both scores are based on less than 10 factors, with integer weights that are summed. These scores should take less than a minute to calculate, once the PET/CT has been completed, and could be automated for even greater ease.

There are some limitations to our study. First, the development of the risk score was carried out using data from an observational study. Inherent limitations do exist with the data and the risk scores due to this.^46^ One of these limitations is the possibility of inaccurate or missed reporting of outcomes. While we did not adjudicate the queried outcomes for this study, we have examined these for prior similar studies and found no systematic bias in the reporting of these in our electronic system.

The outcomes are also limited in that the cause of death was not present in the data. Thus, separating cardiovascular causes of death from other causes was not possible. However, forty to sixty percent of all deaths in prior cardiovascular studies for cohorts with coronary artery disease have been found to be related to cardiovascular diseases.^47–49^ Since our samples have a large percentage with a coronary artery disease history, it is likely that the majority of deaths were cardiovascular related. Another limitation due to observational data is missing data points in the study. To address this, we carried out two types of risk score development, one was with the missing data removed and the other was with the missing data included. In the latter, missing data were given a category of its own in the dataset, and the results between the scores were similar. Accordingly, the development of the risk score proceeded with removing missing factors. Finally, while the use of the test data allowed for an independent set of data for validation of the developed PET risk score, it was pulled from Intermountain Healthcare and not a separate institution. Therefore, the performance in a different patient population is unknown and deserves further investigation.

## Conclusion

A PET/CT 90-day MACE-Revasc risk score and a PET/CT one-year MACE-Revasc risk score were generated that incorporate routinely collected PET/CT results combined with a minimum number of clinical features for simple calculation. These risk scores provide improved prediction over ischemic burden alone and improve the classification, compared to cardiologists, of high-risk patients. The use of these simple PET/CT MACE-Revasc risk scores in an external patient population should be examined as well as the determination of the value of their use in a clinical setting.

## New knowledge gained

The derived PET/CT MACE-Revasc risk scores outperformed ischemic burden alone and the predicted risk of cardiologists. This finding indicates that the combined use of PET/CT data in an easy to calculate risk score may improve clinical assessment and care.

## Supplementary Information

Below is the link to the electronic supplementary material.

Supplementary file1 (DOCX 39 kb)

## Abbreviations

MACE: Major adverse cardiovascular events
PET/CT: Positron emission tomography/computed tomography
MACE-Revasc: MACE and revascularization
TID: Transient ischemic dilation
CAC: Coronary artery calcification
EF: Ejection fraction
MI: Myocardial infarction
ROC: Receiver operating characteristic
CFR: Coronary flow reserve
