# Supplementary material for: A highly predictive cardiac positron emission tomography (PET) risk score for 90-day and one-year major adverse cardiac events and revascularization
Source: J Nucl Cardiol. 2022 Dec 19;30(1):46–58. doi: 10.1007/s12350-022-03028-y (PMC10035554; doi:10.1007/s12350-022-03028-y)
Supplement: Supplementary file 1 — Supplementary file1 (DOCX 39 kb) [file 12350_2022_3028_MOESM1_ESM.docx]

**SUPPLEMENTARY FILE**

**Cardiac PET/CT Acquisition & Reconstruction Parameters**

**Patient preparation**

Patient history covering symptoms, risk factors, diseases, medication, and prior diagnostic or therapeutic procedures was performed. Patients were refrained from taking caffeine-containing substances for at least 24 h and were asked to fast at least 6 h prior to the PET studies; medications that may interfere with the cardiac pharmacological stress agent (e.g., nitrates or beta-blockers) were suspended for ≥24 h.

**Protocol and image acquisition**

All dynamic studies were performed using a 3D PET/CT scanner (Siemens Biograph mCT 20) equipped with lutetium orthosilicate (LSO) scintillation crystals (LSO crystals of size: 4 × 4 × 20 mm); axial field of view (FOV) is 16.4, 22.1*cm with bore diameter at 78 cm. Annihilation photons were acquired in a 425–650 keV energy window. TOF coincidence-timing resolution is 540 ps. Emission images at rest were obtained after ^82^RbCl administration of 20-40mCi (weight based) by an automated system (^82^Sr/^82^Rb Cardiogen Model 1700 generator delivery system). Pharmacological stress was induced with regadenoson (0.4 μg/kg/min) to obtain stress perfusion results. A second injection of radiopharmaceutical (20-40 mCi) was injected ≥10.1 min after the first injection, following 90 seconds of regadenoson intravenous infusion. A list-mode acquisition was started simultaneously with radiopharmaceutical injection for both studies (rest, stress), and dynamic images were reconstructed for deriving myocardial blood flow and reserve measurement. The attenuation maps for rest and stress studies were obtained from the imbedded 20-slice CT scanner adopting a low-dose CT setup (120 kV, 20 quality ref. mAs, 0.5 s per rotation, pitch 1.05). The first CT was acquired after the topogram scan for accurate definition of the axial examination range and just before the rest study; the second CT scan was repeated immediately after the stress acquisition. All stress procedures were supervised by a qualified physician/PA-C with knowledge of pharmacological stress agents and expertise in advanced life support techniques.

**Table S1: Percentage of missing values for candidate factors in training, dev and test data**

| **Variable** | **Training** | **Dev** | **Test** |
| --- | --- | --- | --- |
| BMI | 0.7% | 0.9% | 0.6% |
| CAC present | 0.0% | 0.1% | 0.1% |
| CFR | 0.7% | 0.9% | 0.3% |
| Diabetes | 0.0% | 0.0% | 0.0% |
| EF | 0.9% | 1.0% | 0.9% |
| Inpatient | 3.7% | 4.1% | 3.6% |
| Ischemic burden | 0.0% | 0.0% | 0.0% |
| Smoking history | 0.0% | 0.0% | 0.0% |
| TID | 5.3% | 5.4% | 0.1% |

Note: BMI=body mass index, CAC=coronary artery calcium, CFR= coronary flow reserve, EF=ejection fraction, TID= transient ischemic dilation

**Table S2: Model building candidate factors**

| **Significant factors (p<0.05)** |  | **Strong correlations (r>0.30) and bolded factor has higher AUC** |
| --- | --- | --- |
| Age |  | Age & **gender** |
| BMI |  | **CAC** & history of revascularization |
| CAC |  | History of hyperlipidemia & **age** |
| CFR |  | History of hypertension & **age** |
| Diabetes |  | History of hypertension & **gender** |
| EF |  | History of hypertension & **history of hyperlipidemia** |
| Gender |  | **History of revascularization** & history of MI |
| History of hyperlipidemia |  | **Inpatient** & age |
| History of hypertension |  | **Inpatient** & gender |
| History of myocardial infarction (MI) |  |  |
| History of revascularization |  |  |
| Inpatient |  |  |
| Ischemic burden |  |  |
| Smoking history |  |  |
| TID |  |  |

| **Final factors for selection model** |
| --- |
| BMI |
| CAC |
| CFR |
| Diabetes |
| EF |
| Inpatient |
| Ischemic burden |
| Smoking history |
| TID |
| **Interaction for 90 day** |
| Ischemic burden * TID |
| **Interactions for 1-year** |
| Ischemic burden * TID |
| Ischemic burden * EF |

Note: BMI=body mass index, CAC=coronary artery calcium, CFR= coronary flow reserve, EF=ejection fraction, TID= transient ischemic dilation

**Table S3a: Final logistic regression models and derived score for 90-day MACE-Revasc**

| **Variable, 90-day MACE-Revasc** | **Beta Coefficient** | **Beta Ratio** | **Score** | **P-Value** |
| --- | --- | --- | --- | --- |
| Ischemic burden >10 | 2.2479 | 5.5793 | 6 | <0.0001 |
| Ischemic burden 5-10 | 1.5328 | 3.8044 | 4 | <0.0001 |
| CAC present | 1.4068 | 3.4917 | 3 | <0.0001 |
| CFR < 1.5 | 0.5281 | 1.3107 | 1 | 0.0002 |
| Inpatient | 0.47 | 1.2 | 1 | <0.0001 |
| TID >1.1 | 0.4029 | 1 | 1, ref. score | 0.0014 |
| TID 1.0-1.10* |  |  |  | 0.1104 |
| CFR 1.5-2.3* |  |  |  | 0.9647 |

*Not significant categories of significant variables are included in the model but no beta coefficient or score are reported. Note: CAC=coronary artery calcium, CFR= coronary flow reserve, TID= transient ischemic dilation

**Table S3b: Final logistic regression models and derived score for one-year MACE-Revasc**

| **Variable, One-Year MACE-Revasc** | **Beta Coefficient** | **Beta Ratio** | **Score** | **P-Value** |
| --- | --- | --- | --- | --- |
| Ischemic burden >10 | 1.4946 | 6.00 | 6 | <0.0001 |
| CAC present | 1.1421 | 4.5849 | 5 | <0.0001 |
| Ischemic burden 5-10 | 0.9427 | 3.7844 | 4 | <0.0001 |
| CFR < 1.5 | 0.8533 | 3.4255 | 3 | <0.0001 |
| Current smoker | 0.481 | 1.93 | 2 | 0.0001 |
| Inpatient | 0.54 | 2.20 | 2 | <0.0001 |
| TID 1.0-1.10 | 0.3587 | 1.44 | 1 | <0.0001 |
| Diabetic | 0.3526 | 1.42 | 1 | <0.0001 |
| TID >1.1 | 0.3163 | 1.26 | 1 | 0.0003 |
| CFR 1.5-2.3] | 0.2491 | 1 | 1, ref. score | 0.0039 |
| Obese (BMI >30) | -0.4274 | -1.72 | -2 | <0.0001 |
| Smoker Former* |  |  |  | 0.2917 |
| Smoker Unclassified Status * |  |  |  | 0.1055 |
| Underweight (BMI<18.5)* |  |  |  | 0.3484 |
| Overweight (BMI 25-30)* |  |  |  | 0.0685 |

*Not significant categories of significant variables are included in the model but no beta coefficient or score are reported. Note: BMI=body mass index, CAC=coronary artery calcium, CFR= coronary flow reserve, TID= transient ischemic dilation

**Table S4: Test Set Area Under Curve (AUC) for PET/CT Risk Scores compared to individual PET result values**

|  | **Area Under Curve (AUC)** | | | |
| --- | --- | --- | --- | --- |
| **MACE-Revasc** | **PET/CT risk Score** | **Ischemic Burden** | **CFR** | **Summed Stress** |
| **90-day** | 0.85 | 0.83 | 0.69*** | 0.83 |
| **One-year** | 0.80 | 0.76*** | 0.69*** | 0.78** |

Note: CFR= coronary flow reserve, * p<0.05, ** p<0.01, *** p<0.001 for comparison with PET risk score

**Table S5: Net Reclassification rates for the PET/CT risk score compared to cardiologist assessment.**

|  | **90-Day MACE-Revasc** | **One-Year MACE-Revasc** |
| --- | --- | --- |
| Events moving up | 120 | 181 |
| Events moving down | 11 | 11 |
| Total Events | 367 | 640 |
|  |  |  |
| Non-Events moving up | 278 | 291 |
| Non-Events moving down | 13 | 16 |
| Total Nonevents | 4682 | 4409 |
|  |  |  |
| Rate of Events moving up (%) | 32.7 | 28.3 |
| Rate of Events moving down (%) | 3.0 | 1.7 |
| Rate of Non-Events moving down (%) | 0.3 | 0.4 |
| Rate of Non-Events moving up (%) | 5.9 | 6.6 |
|  |  |  |
| NRI Events | 0.30 (0.25,0.35) | 0.27 (0.23,0.30) |
| NRI Non-Event | -0.06 (-0.06, -0.05) | -0.06 (-0.07, -0.05) |
| NRI (total) | 0.24 (0.19, 0.30) | 0.20 (0.16, 0.25) |

*** categorization of score was low/moderate vs. high**

**Table S6:** **Test Set Area Under Curve (AUC) for PET/CT Risk Scores compared to individual PET result values for MACE without revascularization**

|  | **Area Under Curve (AUC)** | | | |
| --- | --- | --- | --- | --- |
| **MACE** | **PET/CT risk Score** | **Ischemic Burden** | **CFR** | **Summed Stress** |
| **90-day** | 0.72 | 0.67** | 0.67* | 0.70 |
| **One-year** | 0.74 | 0.65*** | 0.69*** | 0.69** |

Note: CFR= coronary flow reserve, * p<0.05, ** p<0.01, *** p<0.001 for comparison with PET risk score
